# The impact of the English national health inequalities strategy on inequalities in mortality at age 65: a time-trend analysis

**DOI:** 10.1093/eurpub/ckae081

**Published:** 2024-05-07

**Authors:** Natalie C Bennett, Paul Norman, Viviana Albani, Andrew Kingston, Clare Bambra

**Affiliations:** Population Health Science Institute, Faculty of Medical Sciences, Newcastle University, Newcastle upon Tyne, UK; Sheffield Methods Institute, Faculty of Social Sciences, The University of Sheffield, Sheffield, UK; School of Geography, Faculty of Environment, University of Leeds, Leeds, UK; Population Health Science Institute, Faculty of Medical Sciences, Newcastle University, Newcastle upon Tyne, UK; Population Health Science Institute, Faculty of Medical Sciences, Newcastle University, Newcastle upon Tyne, UK; Population Health Science Institute, Faculty of Medical Sciences, Newcastle University, Newcastle upon Tyne, UK

## Abstract

**Background:**

During the 1997–2010 Labour government, several policies were implemented to narrow health inequalities as part of a national health inequalities strategy. Many of these policies are likely to have had a disproportionately large impact on people aged 65 and over. We aimed to understand the association between the health inequalities strategy period and inequalities in mortality at age 65–69.

**Methods:**

We use population at risk and mortality data covering 1991–2019 to calculate mortality rate at age 65–69 at the Local Authority level. We use the 2019 Index of Multiple Deprivation to examine geographical inequalities. We employ segmented linear regression models with marginal spline terms for the strategy period and interact these with an indicator of deprivation to understand how inequalities changed before, during and after the strategy. The reporting of this study adheres to STROBE guidelines.

**Results:**

Mortality rates in each deprivation quintile improved continuously throughout the period of study. Prior to the programme (1991–9) there was no significant change in absolute inequalities. However, during the strategy (2000–10) there was a significant decrease in absolute inequalities of −9.66 (−17.48 to −1.84). The period following the strategy (2011–19) was associated with a significant increase in absolute inequalities of 12.84 (6.60 to 19.08). Our results were robust to a range of sensitivity tests.

**Conclusion:**

The English health inequalities strategy was associated with a significant reduction in absolute inequality in mortality age 65–69. Future strategies to address inequalities in ageing populations may benefit from adopting a similar approach.

## Introduction

Countries in the Global North are experiencing population ageing with significant impacts on health care, labour markets and social security systems.^1^ England is a key example, with over 18% of the population now aged 65 and over.^2^ Research suggests that improvements in life expectancy at age 65 in England have slowed since around 2011.^3^ There are inequalities in health by area-level deprivation and region across England, with people aged over 65 living in areas in the most deprived decile on average living between 8 (women) and 9.7 (men) fewer years than those living in areas in the least deprived decile.^4^ Similarly, there are inequalities in multimorbidity and healthy life expectancy.^5^ Studies point to a widening of inequalities in life expectancy,^6–8^ and in mortality in age groups over 40 since 2011.^9^

In response to this rising health inequality, the UK government set a target to narrow the gap in healthy life expectancy between the ‘richest and poorest areas’ of England by 2030 and to raise overall life expectancy by five years by 2035.^10^ The last time the UK government had targets to reduce heath inequalities was under the 2000–10 English health inequalities strategy.^11^^,^^12^

In 1997, a Labour government was elected against a backdrop of rising health, social, economic and regional inequalities.^13^ Following the 1998 Acheson Inquiry recommendations, a new national health inequalities strategy was implemented across government.^14^^,^^15^ The multifaceted strategy included large increases in public spending on various social programmes, reductions in child and old age poverty rates, the introduction of the national minimum wage, area-based interventions like Health Action Zones, public service agreement targets and a substantial increase in expenditure on the NHS.^16^^,^^17^ In addition, a cornerstone of Labour’s manifesto was tackling pensioner poverty and providing a ‘decent and secure’ income for pensioners.^18^ Importantly, several of the interventions implemented during the strategy had specific relevance to those aged over 65:

An increase in the value of state pensions (via improved value of the basic universal state pension and a Minimum Income Guarantee/Pension Credit for the poorest pensioners).^19^^,^^20^;
Winter fuel payments (from 1997) for everyone aged over 60; free TV licences for the over 75s (2000–20); and free bus passes for the over 60s (since 2007).^20^;
An increase in NHS funding overall (disproportionately benefitting older age groups given their higher health needs) and the addition of a ‘health inequalities weighting’ to the way NHS funds were geographically distributed, with more allocated to more deprived areas.

The national health inequalities strategy ended with Labour’s loss in the 2010 general election, followed by a succession of Conservative-led governments (2010–15; 2015–17; 2017–19; 2019–24). The government change led to the reversal of many key facets of the strategy under a policy of austerity. Public expenditure was reduced in response to the 2007/8 Global Financial Crisis with the intention of reducing the national deficit, including reductions to the NHS budget, cuts to education and social care and steep reductions across the social welfare system.^17^^,^^21^^,^^22^

To improve our knowledge of what works to improve healthy ageing and reduce health inequalities amongst older age groups in England, and aid international policymakers and researchers in other territories with ageing populations, this article examines the impacts of the 2000–10 national health inequalities strategy on area-level inequalities in mortality rates at age 65–69 years.

## Methods

### Data

We use mortality rate at age 65–69 as our outcome, based on our interest in the impacts of the strategy on older age groups and pensioners. In England, pension age was approximately age 65 over the strategy period. In addition, many elements of the strategy (see those described above) had particular relevance to those of pensionable age, meaning this group should have seen more of the effects of the strategy than the younger five-year age band for example. However, we also provide analyses of the age bands either side of our main age band of interest and present the results of these models in the Supplementary appendix. Mid-year mortality data (undifferentiated by sex) for the 65–69 age group were accessed from the Office for National Statistics’ (ONS) (the UK’s official department for the collection and maintenance of national statistics) tables covering the overlapping periods 1991–2017 and 2010–21, and aggregated into one file.^23^ Mid-year population estimates (1991–2019) from the ONS were used to capture populations at risk.^24^ The time series starts in 1991 (the earliest year available) and ends in 2019, avoiding the mortality impacts of the COVID-19 pandemic. Both mortality and population at risk data were provided at the Lower Super Output Area (LSOA) level (a census geography typically used to refer to neighbourhoods) and aggregated to 2021 Lower Tier Local Authority (LTLA) boundaries for this analysis. LTLAs represent local political units at which decisions are made surrounding the provision and maintenance of local public services. There are 309 LTLAs in England, representing a population size of 50 000–600 000. We aggregate to this scale in order to ensure that the number of deaths in the 65–69 age group was large enough to provide reliable mortality rate estimates, as well as to protect confidentiality. Furthermore, as LTLAs represent local political boundaries, modelling deaths at this scale with robust standard errors allows us to control for any between LTLA variation which might be a result of differences in LTLA specific implementation of elements of the strategy. Mortality rate at age 65–69 was estimated as the age-specific mortality of a given LTLA in a given year, divided by the population at risk within that LTLA, multiplied by 100 000.

Area-level deprivation was measured using England’s official measure of area deprivation, the Index of Multiple Deprivation (IMD).^25^ The IMD has been in use since 2000, and therefore, covers the whole of the strategy period. The IMD is comprised of seven weighted domains, sourced largely from administrative data. The most recent edition of the IMD data (2019) was employed^25^ so that our findings are most relevant to current political debates and understandings of area deprivation. Generally, research suggests that area-level deprivation changes very little over time, especially concerning the most deprived areas.^26^^,^^27^ Furthermore, research analysing the same policy period found that the year of deprivation measure used made no difference to relationships with their outcome (infant mortality).^16^ We estimate LTLA deprivation scores by calculating the average rank of IMD scores in the LSOA comprising each LTLA, weighted by 2011 LSOA population size.^28^ From this LTLA deprivation average rank, we produce quintiles which we employ to understand how deprivation-based geographical inequalities changed over the policy period.

### Analyses

We aimed to assess the trend in inequalities in mortality rates at age 65–69 before, during and after the policy period, between the most deprived areas and the rest of England. We hypothesized that, during the period of interest, there would be a reduction in the gap between the most deprived areas and the rest of England.

We present an initial descriptive graph showing the trend in mortality rate at 65-69 across all five quintiles, followed by graphs to show relative and absolute difference in mortality rate at age 65–69. We calculate absolute inequality as the absolute difference between the most and least deprived quintile of LTLAs, and relative inequality as the most deprived quintile divided by the least deprived. We then follow previous studies in analysing inequalities for the 20% most deprived LTLAs, compared with the rest of England (the other 80%).^16^^,^^29^ To quantify the change in absolute inequality, we ran a fixed effects segmented linear regression model with marginal spline terms to mark the start and end of the policy period, with cluster robust standard errors. We chose the year 2000 for the first spline and the year 2010 for the second spline. We do not include a lag in the strategy period start in the analysis presented in the paper (though robustness tests including lagged effects are included in the Supplementary tables S3–S6). The introduction of some of the measures which could be thought of as part of the strategy began from as early as 1997, when the new Labour government was elected. Taking 2000 as the first spline therefore accounts for the phased implementation of this multifaceted strategy. We included an interaction between the spline terms and a binary indicator of the most deprived 20% of LTLAs to produce an estimate of this inequality for each period. Coefficients from the model can be interpreted as indicating the average trend in absolute inequality in mortality rate at age 65–69 for each time segment of the model; before, during and after the health inequalities strategy period. More detail on the model specification is provided in the appendix (Supplementary table S1). We took a theory-driven approach to selecting the dates for the spline terms in the model, based on previous work evaluating the strategy and our knowledge of how these policies may have impacted the mortality rates of people 65 and over.^16^ We present the results of the model analysing the full health inequalities strategy period (2000–10) within the paper. Additional robustness tests—described below—are presented in the appendix (Supplementary tables S1–S9).

### Robustness tests

We performed various tests to assess the robustness of our analyses. We tested linear spline terms (Supplementary table S1), rather than marginal, to assess the interval slopes themselves, rather than the change in slope from the previous period. We ran a random effects model (Supplementary table S2) to examine whether modelling heterogeneity by LTLA improved our estimates. Different date cut-points were operationalized to assess whether our results held using years proximal to the strategy period and other potentially relevant policy periods (Supplementary tables S3–S6). We additionally modelled two younger age bands (55–59 and 60–64) (Supplementary tables S7 and S8) and one older age band (70–74) (Supplementary table S9).

This study adheres to the STROBE reporting guidelines for observational studies (see Supplementary appendix).

## Results

### Descriptive analyses

**Figure 1:**
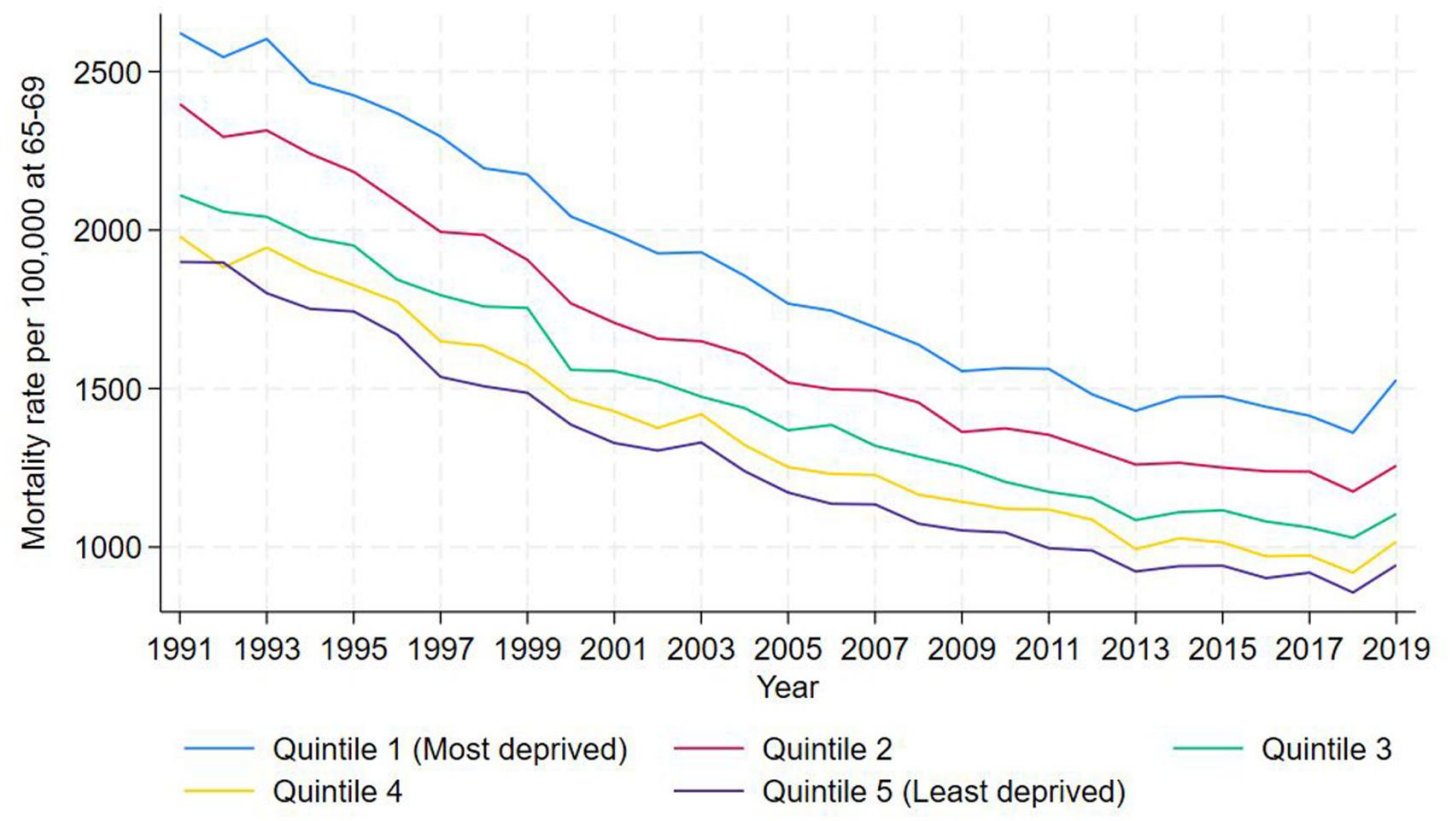
Mortality rate at age 65–69 for each of the five quintiles of LTLA deprivation, 1991–2019

Overall mortality rates in England at age 65–69 have been decreasing since the beginning of the time series. Figure 1 shows this trend across all deprivation quintiles and demonstrates consistent improvement from around 1991 until around 2013, after which improvements slowed. Mortality rate at age 65–69 decreased from 2593.08 per 100 000 (SD 348.69) in 1991 to 1520.63 (SD 214.39) in 2019 (a decrease of 1072.45) for the most deprived quintile, and from 1899.67 (SD 222.04) in 1991 to 943.72 (SD 150.33) in 2016 for the least deprived quintile (a decrease of 955.95).

**Figure 2:**
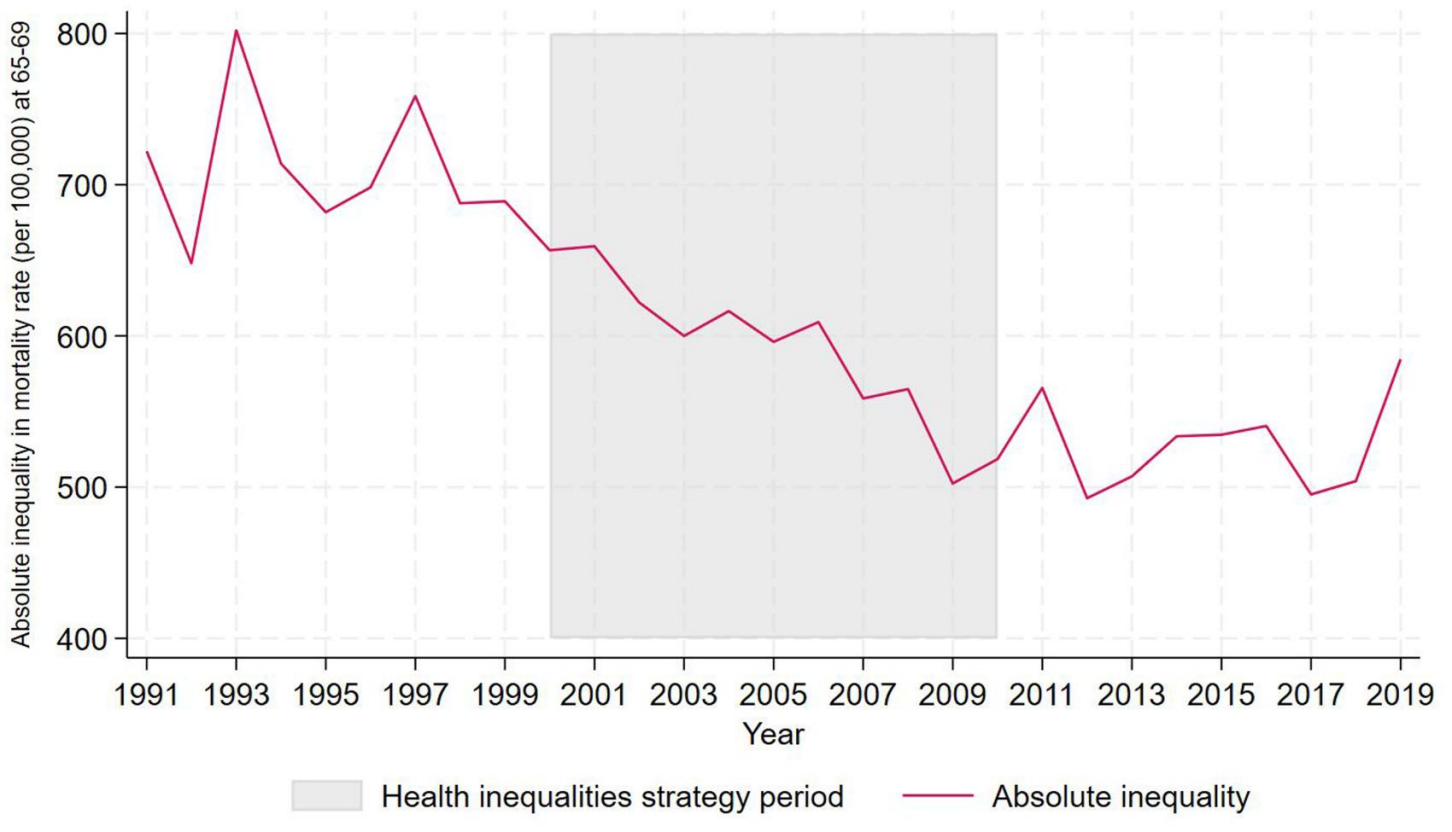
Absolute inequality in mortality rates at age 65–69, 1991–2019: LTLAs in IMD Quintile 1 compared with Quintile 5, 1991–2019

**Figure 3:**
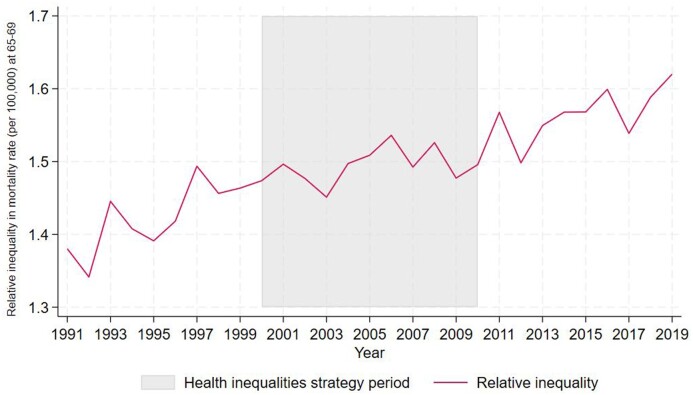
Relative inequality in mortality rate at age 65–69: LTLAs in IMD Quintile 1 compared with Quintile 5, 1991–2019

We show trends in mortality rate inequalities in both absolute and relative terms in accordance with previous research and guidance on the analysis of health inequalities.^30^^,^^31^  Figures 2 and 3 show these trends, highlighting the health inequalities strategy period. Figure 2 shows absolute inequalities in mortality rate between the most and least deprived areas. The figure shows a general downward trend from around 1999 in absolute inequalities, with the smallest difference between the most and least deprived areas observed in 2012, after which inequalities fluctuate. Prior to the strategy, absolute inequalities decreased by 33.20 per 100 000 slightly from 722.22 in 1991–689.02 in 1999. During the strategy, relative inequalities decreased by 138.15 per 100 000 from 656.66 in 2000, to 518.51 in 2010. However, absolute inequality began to increase after the strategy, by 18.96 per 100 000, from 565.60 in 2011 to 584.56 in 2019.

Figure 3 shows relative inequality between mortality rate at age 65–69 for the most compared with the least deprived quintiles, whereby higher numbers indicate larger inequalities in mortality rate between the quintiles. The figure shows a general increase in relative mortality inequalities across the whole time series. Prior to the strategy, relative inequality increased from 1.38 in 1991 to 1.46 in 1999. During the strategy, relative inequalities increased slightly from 1.47 in 2000, to 1.49 in 2010. Relative inequalities continued to increase during the post strategy period, reaching 1.62 by 2019.

### Modelling results

Results from the fixed effects, segmented linear regression model, showing inequalities before, during and after the policy period are presented in table 1. The estimate for the period before the strategy was not statistically significant (−1.22, 95% CI −7.68 to 5.24). During the health inequalities strategy period, absolute inequalities between the 20% most deprived areas and the rest of England decreased significantly by an average of −9.66 per 100 000 (95% CI −17.48 to −1.84) per year. In the period after the strategy, inequalities between the 20% most deprived and the rest of England increased significantly by an average of 12.84 per 100 000 (95% CI 6.60 to19.08) per year.

**Table 1:** Absolute inequalities in mortality rate at age 65–69 between the most deprived 20% of LTLAs compared with the rest of England before, during and after the policy strategy period

Period	Annual change in the absolute difference between the most deprived 20% of LTLAs and the rest of England. Coefficient (95% CI)
Before (1991–9)	−1.22 (−7.68 to 5.24)
During (2000–10)	−9.66 (−17.48 to −1.84)
After (2011–19)	12.84 (6.60 to 19.08)

### Robustness analyses results

Modelling using linear, rather than marginal spline terms, produced a similar strategy estimate (though with a much narrower confidence interval) (−10.88, 95% CI −14.59 to −7.18), but a much smaller post-strategy estimate (1.96, 95% CI −2.62 to 6.54) (Supplementary table S1). All four-time period variations (Supplementary tables S3–S6) produced broadly similar effect estimates (in terms of direction, size and confidence) for each of the time segments, except for the Labour Government policy period. In this model, the ‘intervention’ segment returns an estimate of −13.34 (95% CI −23.37 to 3.31), the largest estimated reduction in absolute inequalities of any of the alternative models for the age 65–69 age group.

We find similar trends in terms of the estimate direction and statistical significance for each of the three alternative age bands tested. However, for the age group immediately below pensionable age (60–64) the widening of absolute inequalities in the period after the health inequalities strategy is much smaller than that of the 65–69 group. For the age group immediately following the first pensionable age group (70–74) the absolute inequalities estimate for the strategy period is larger (though with more uncertainty) than the 65–69 group, but has a similar estimate for widening inequalities after this period. Ultimately, we found a narrowing of absolute inequalities during the ‘intervention’ segment in all of the robustness tests we conducted.

## Discussion

There have been several previous evaluations of the impact of the English national health inequalities strategy on reducing health inequalities. A recent systematic review concluded that ‘*the health inequalities strategy led to a reduction in absolute inequalities in life expectancy, mortality, infant mortality and major causes of death [and] … there seemed to be a narrowing of relative inequalities in at least life expectancy and infant mortality*’*).*^32^ However, the current evidence base of such a large-scale and long-term strategy remains relatively small. A key evidence gap is the effect of the strategy on health inequalities amongst older people. As noted previously, several policies implemented during the strategy period were likely to have had a disproportionately large and potentially beneficial effect on the health of older people—particularly those in the most deprived areas.

Our results suggest that absolute inequalities in mortality rate at age 65–69 between the most deprived 20% of LTLAs and the rest of England reduced significantly during the health inequalities strategy period. However, this reduction in inequalities was not sustained in the period following the strategy when absolute inequalities significantly increased. Importantly, the increase in pensions initiated during the strategy led to a substantial decline in poverty rates amongst pensioners from 26% in 1997–17% in 2010.^33^ It was followed by the pensions ‘Triple Lock’ (whereby the state pension was increased in line with wages, inflation or 2.5%—whichever was highest) after 2010 and other pre-2010 policies (such as the winter fuel allowance) were also maintained. However, pensioner poverty still rose to 19% in 2020.^33^ While the reduction in pension poverty during the strategy period may have contributed to the reductions in absolute inequalities in mortality; other aspects of the strategy may also have played a role, such as improved funding for the NHS. Indeed, austerity measures (and the associated large reductions in social care budgets and below inflation increases to NHS budgets) implemented from 2010 onwards have been associated with declines in the health of older groups, such as rising mortality rates for those over 65 and 85, and shortened life expectancy at 65.^34–36^ This likely in part explains the widening of inequalities in the post health inequalities strategy period. Furthermore, our robustness analysis—which found a similar reduction in inequalities in the pre-pension age group—also points towards factors other than pensioner incomes as driving the reduction in inequalities during the strategy period in older age groups. As policy discourse in the UK debates the possible termination of the ‘Triple Lock’ and the future of pensioner income protection more generally, proposals for change must address both widening inequalities and prioritizing the guaranteed receipt of sufficient support to those who most need it. This has implications for other countries, such as Spain or Portugal, where pensioner poverty rates are high.^1^

It is important to note that, while we find a reduction in absolute inequalities during the strategy, relative inequalities increased across the data series. The phenomenon of opposite trends in absolute versus relative inequalities is well known in the health inequalities literature.^37^ Mackenbach notes that reductions in relative inequalities are rarely observed, because in cases where background risk is low, relative risks for a chosen exposure will typically be higher. Mackenbach goes on to state that ‘In a context of rapidly declining mortality rates, it is extremely difficult to reduce relative inequalities in mortality’ (p.185). Our findings of a reduction only in absolute inequalities therefore echoes the findings of other literature examining inequalities in mortality rates.

### Strengths 

This analysis has several strengths, including benefitting from a long data series, and the use of LTLA-level data, allowing us to estimate within-LTLA effects for each time period. We also employ open access data and make our code available to support reproducibility efforts. To our knowledge, this is the first study to explicitly examine the impact of the English health inequalities strategy period on older people. The results of our analyses were robust to a range of tests we subjected them to, with a reduction in absolute inequalities found during the strategy period in all.

### Limitations

Several limitations should also be considered. Firstly, this research is ecological in nature, and therefore, the findings pertain to LTLAs and not individuals or sub-areas within them. We also assume that LTLA deprivation quintile is static over time in our analysis. Although research suggests that area-deprivation generally changes little over time,^38^ with 70% of LTLAs remaining in the same quintile over time and 78% of the most deprived persisting at that level,^26^ this is likely an oversimplification. However, the results of robustness tests by a similar paper suggest that the choice of deprivation measure is unlikely to substantially affect the results of this analysis.^16^ In addition, the coefficients estimated by our models are relatively small and the confidence intervals are relatively wide. However, the consistency of findings of a reduction in inequalities across all our models suggests inequalities did decrease during the strategy period. Caution is advised when interpreting these model estimates. Other political and economic events occurred during the time series, including relative economic stability during the strategy period and a global recession from 2007–8. Finally, we do not stratify our analyses by gender, despite known inequalities in mortality, life expectancy and pensions between men and women in England.^39^^,^^40^ Future research should seek to understand inequalities in mortality by gender over this period and assess any specific role for pensions.

## Conclusion

Our analysis suggests that English national health inequalities strategy was associated with a decrease in absolute geographical inequalities in mortality at age 65–69, whereas the following period was associated with a significant worsening of inequalities. Future efforts to reduce heath inequalities among older age groups should prioritize both sufficient income levels and the provision of adequate funding of health and social services, especially in the most deprived areas.

## Supplementary Material

ckae081_Supplementary_Data

## Data Availability

The data employed in this analysis are freely available to download from the locations linked in the appropriate references. The code for the data preparation, analyses and graphs will be made available in the Supplementary material.
