# Supplementary material for: The impact of the English national health inequalities strategy on inequalities in mortality at age 65: a time-trend analysis
Source: Eur J Public Health. 2024 May 7;34(4):660–5. doi: 10.1093/eurpub/ckae081 (PMC11299195; doi:10.1093/eurpub/ckae081)
Supplement: ckae081_Supplementary_Data [file ckae081_supplementary_data.zip › ckae081_Supplementary_Data/ejph-2024-01-om-0027-File005.docx]

Appendix materials

A1 – Model specification

MR*_Quint_*,*_t_* = 𝛼_0_ + 𝛽_1_𝑡_1_ + 𝛽_2_𝐷𝑒𝑝rived + 𝛽_3_𝐷𝑒𝑝rived ∗ 𝑡_1_ + 𝛽_4_𝑡_2_ + 𝛽_5_𝐷𝑒𝑝rived ∗ 𝑡_2_ + 𝛽_6_𝑡_3_ + 𝛽_7_𝐷𝑒𝑝rived + 𝑢 *_Quint_*,*_t_* + 𝜀 *_Quint_*,*_t_*

Where MR*_Quint_*,*_t_* is the average mortality rate (MR) in a quintile of the IMD (*_Quint_*) at year (*_t_*). 𝛼_0_ denotes the constant term in the model. 𝑡_1,_ 𝑡_2, and_ 𝑡_3_ denote the marginal spline terms used in the final model presented in the paper. These can be substituted to denote linear spline terms in our alternate model presented in A1. The binary indicator for the most deprived quintile is denoted with 𝐷𝑒𝑝rived. The time-invariant error term for the deprivation quintiles is denoted by 𝑢 *_Quint_*,*_t_* while the random error term is denoted by 𝜀 *_Quint_*,*_t_*.

Table A1

Linear spline terms

| Period | Annual change in the absolute difference between the most deprived 20% of Local Authorities and the rest of England.  Coeff (95% CI) |
| --- | --- |
| Before (1991-1999) | -1.22 (-7.68 to 5.24) |
| During (2000-2010) | -10.88 (-14.59 to -7.18) |
| After (2011-2019) | 1.96 (-2.62 to 6.54) |

Table A2

Random effects model

| Period | Annual change in the absolute difference between the most deprived 20% of Local Authorities and the rest of England.  Coeff (95% CI) |
| --- | --- |
| Before (1991-1999) | -1.22 (-7.66 to 5.22) |
| During (2000-2010) | -9.66 (-17.45 to -1.87) |
| After (2011-2016) | 12.84 (6.62 to 19.06) |

Table A3

Time period variation 1 (The Labour government period)

| Period | Annual change in the absolute difference between the most deprived 20% of Local Authorities and the rest of England.  Coeff (95% CI) |
| --- | --- |
| Before (1991-1996) | 3.65 (-5.72 to 13.03) |
| During (1997-2010) | -13.34 (-23.37 to -3.31) |
| After (2011-2019) | 11.08 (5.22 to 16.94) |

Table A4

Time period variation 2 (1 year lag of whole Health Inequalities Strategy (HIS) period)

| Period | Annual change in the absolute difference between the most deprived 20% of Local Authorities and the rest of England.  Coeff (95% CI) |
| --- | --- |
| Before (1991-2000) | -2.22 (-7.99 to 3.54) |
| During (2001-2011) | -8.55 (-15.76 to -1.33) |
| After (2012-2019) | 14.33 (7.53 to 21.13) |

Table A5

Time period variation 3 (lagged start of HIS)

| Period | Annual change in the absolute difference between the most deprived 20% of Local Authorities and the rest of England.  Coeff (95% CI) |
| --- | --- |
| Before (1991-2000) | -1.94 (-7.76 to -3.88) |
| During (2001-2010) | -9.57 (-17.06 to -2.09) |
| After (2011-2019) | 13.74 (7.25 to 20.24) |

Table A6

Time period variation 4 (lagged end of HIS)

| Period | Annual change in the absolute difference between the most deprived 20% of Local Authorities and the rest of England.  Coeff (95% CI) |
| --- | --- |
| Before (1991-1999) | -1.51 (-7.92 to 4.91) |
| During (2000-2011) | -8.78 (-16.39 to -1.17) |
| After (2012-2019) | 13.59 (6.99 to 20.19) |

Table A7

Age 55-59

| Period | Annual change in the absolute difference between the most deprived 20% of Local Authorities and the rest of England.  Coeff (95% CI) |
| --- | --- |
| Before (1991-1999) | 0.44 (-3.24 to 4.12) |
| During (2000-2010) | -5.54 (-10.58 to -0.51) |
| After (2011-2016) | 3.60 (0.24 to 6.95) |

Table A8

Age 60-64

| Period | Annual change in the absolute difference between the most deprived 20% of Local Authorities and the rest of England.  Coeff (95% CI) |
| --- | --- |
| Before (1991-1999) | 1.82 (-2.80 to 6.43) |
| During (2000-2010) | -9.32 (-15.54 to -3.09) |
| After (2011-2016) | 5.40 (0.53 to 10.27) |

Table A9

Age 70-74

| Period | Annual change in the absolute difference between the most deprived 20% of Local Authorities and the rest of England.  Coeff (95% CI) |
| --- | --- |
| Before (1991-1999) | 5.63 (-3.21 to 14.48) |
| During (2000-2010) | -15.78 (-27.62 to -3.94) |
| After (2011-2016) | 9.48 (0.09 to 18.88) |
